# Detection of left ventricular diffuse fibrosis with quantitative T1 mapping in patients with paroxysmal atrial fibrillation

**DOI:** 10.1186/1532-429X-15-S1-P117

**Published:** 2013-01-30

**Authors:** Raymond H Chan, Murilo Foppa, Sebastian Weingärtner, Kraig V Kissinger, Beth Goddu, Warren J Manning, Reza Nezafat

**Affiliations:** 1 Beth Israel Deaconess Medical Center, Chestnut Hill, MA, USA

## Background

Background: Quantitation of T1 relaxation times (T1 time) is a novel non-invasive CMR technique which can detect both interstitial and replacement fibrosis. T1 changes consistent with interstitial fibrosis have been described in conditions associated with left ventricular (LV) remodeling and systolic dysfunction, including chronic mitral regurgitation and aortic stenosis. Atrial fibrillation (AF) is also associated with left ventricular (LV) remodelling. With AF, fibrosis develops in the atrium in AF, but it is unknown whether the same processes occur in the left ventricle.

Hypothesis: We hypothesized that patients with paroxysmal AF would demonstrate CMR evidence of LV fibrosis, exhibiting decreased T1 times compared with control patients.

## Methods

We prospectively enrolled 35 patients referred for a contrast-enhanced cardiac MR scan at 1.5T for clinical purposes and were in sinus rhythm during the time of the scan. Seventeen patients had paroxysmal AF, while 18 had no CMR evidence of structural or valvular abnormalities (controls). A Modified Look-Locker Inversion Recovery (MOLLI) sequence was acquired before and after bolus administration of 0.1 mmol/kg gadobenate dimeglumine (Multihance) for 3 short-axis slices. T1 times were measured in the entire LV myocardium of the 3 slices, and the values for the 3 slices were then averaged to reflect the overall T1 time of the entire LV.

## Results

The 35 patients included in the analysis comprised of 60% men (age 56±14 years). Baseline characteristics were similar between AF (n=17) and control (n=18) groups (age 59±12 vs 53±16 yrs, p=0.27; ejection fraction (EF) 56±13 vs 60±5%, p=0.29; LV mass index 57±17 vs 56 ± 16 g/m^2^, p=0.88, end diastolic volume 173±59 vs 163 ±44 ml, p=0.57, stroke volume 90±17 vs 97±24 ml, p=0.33). Pre-contrast T1 times were not significantly different between those with AF and the control group (1028 ± 67 vs 1012 ± 37 ms, p=0.47). However, post-contrast T1 times were significantly lower in those with AF (395 ± 69 vs 447 ± 67 ms, p=0.04). After controlling for EF and time after contrast injection, patients with AF continued to exhibit significantly lower T1 times compared with controls (adjusted difference= -46 ± 21 ms, p=0.04).

## Conclusions

Atrial fibrillation is associated with a significantly lower post-contrast T1 time, without an apparent decrease in the pre-contrast T1 time. These findings suggest post-contrast T1 mapping may be more sensitive in detecting early LV remodeling associated with paroxysmal atrial fibrillation.

## Funding

Nil

